# Bit Error Rate Closed-Form Expressions for LoRa Systems under Nakagami and Rice Fading Channels

**DOI:** 10.3390/s19204412

**Published:** 2019-10-12

**Authors:** Claudio Ferreira Dias, Eduardo Rodrigues de Lima, Gustavo Fraidenraich

**Affiliations:** 1 Department of Digital Hardware Design, Instituto Eldorado, Eldorado, Alan Turing - 275, Campinas 13083-898, Brazil; eduardo.lima@eldorado.org.br; 2 Department of Communications, University of Campinas, Albert Einstein - 400, Campinas 13083-852, Brazil; gf@decom.fee.unicamp.br

**Keywords:** LPWAN, Internet of Things, MIMO, Nakagami, Rayleigh, Rice

## Abstract

We derive exact closed-form expressions for Long Range (LoRa) bit error probability and diversity order for channels subject to Nakagami-*m*, Rayleigh and Rician fading. Analytical expressions are compared with numerical results, showing the accuracy of our proposed exact expressions. In the limiting case of the Nakagami and Rice parameters, our bit error probability expressions specialize into the non-fading case.

## 1. Introduction

Low Power Wide Area Networks (LPWAN) are the new trend in the development of wireless communication technologies in the era of the Internet of Things (IoT) [1]. Long Range (LoRa) [2] technology has awakened the interest of the research community due to its growing usage for LPWAN deployments [2,3,4,5].

As clearly stated in [6,7,8,9], LoRa technology is mainly based on the features of the chirp spread spectrum (CSS) modulation. In [10], it establishes a more solid mathematical representation of the modulation/demodulation process addressing the bit error rate (BER) performance for AWGN and frequency selective channels. In [11], the BER was derived assuming CSS modulation and the use of moment matching to obtain analytical BER approximations for AWGN and Rayleigh scenarios. The BER performance under the perspective of orthogonal signaling described in [12] can be used to assess the performance of chirp spread spectrum modulation, as pointed out in [10].

The primary motivation of this investigation is to find new expressions for the bit error rate of chirp spread spectrum (CSS) modulation in the essential fading scenarios. For instance, Rayleigh statistics models well the signal power in a multipath environment [13]. On the other hand, Rice appropriately models multipath in situations with a dominant line-of-sight signal. In complex dense urban environments, we expect all kinds of small-scale fading, i.e., Ricean fading for strong, static links, and a smooth transition from Rayleigh fading down to Double-Rayleigh fading for moving links. A mathematically convenient method to approximate these distributions is by using the Nakagami distribution [14]. The use of Nakagami mathematical method is broad and can describe the amplitude of a received signal after maximum ratio diversity combining, since the *k*-branch MRC with Rayleigh-fading signals results in a Nakagami with m=k. In this way, it is possible to use the Nakagami-*m* distribution to investigate the use of multiple antennas (MIMO) in LoRa gateway (or LoRa base station). The Nakagami distribution is also the best match for many fading measured data, as related in [15,16,17].

The main contribution of our work is the derivation of exact closed-form expressions to calculate the CSS BER performance in diverse scenarios such as Rayleigh, Rician, and Nakagami fading channels. We believe that our main results extend the knowledge in this topic by including two new scenarios: the performance analysis under the Nakagami-*m* case and the Rice case. As it is well known in the literature, as the Nakagami parameter m=1, or the Rice parameter κ=0, our results specialize into the Rayleigh case. Also, the authors in [11] provided an approximate expression for the Rayleigh case, whereas we have provided an exact and elegant result.

We organize the remainder of this paper as follows: Section 2 refers to the underlying system model; Section 3 refers to the exact bit error probability performance derivation. Section 4 confirms the accuracy of the proposed exact expressions. Finally, Section 5 points out the main aspects of our achievements.

## 2. System Model

**Figure 1:**
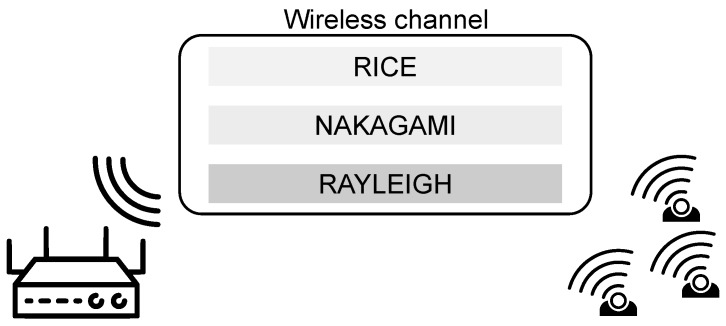
System Model.

Figure 1 illustrates the system model in which the designer can choose one out of three options of essential fading environments. LoRa devices use shift chirp modulation scheme for communication. The modulation consists of a signal which chirps within a frequency range in a given period. It is also known as spread spectrum modulation, and different symbols can be composed by varying bandwidth and spread spectrum parameters. The spreading factor (SF) determinates the number of samples within a symbol duration such that $T_{s}=\frac{2^{SF}}{B}$, where *B* is the signal bandwidth and SF∈{6,7,…,12} which are the values available for typical commercial devices [18].

The encoder maps every SF bits to a symbol $s_{k}$, such that $k\in \{0,1,\ldots ,2^{SF}-1\}$. We then understand that *k* is related to a $f_{k}=B\frac{k}{2^{SF}}$ offset frequency. Each chirp signal has a specific starting frequency, and it wraps around bandwidth *B* as long as frequency keeps increasing. The chirping rate is given by $\frac{B^{2}}{2^{SF}}$ and signal decodes according to the position offset of $f_{k}$. The transmitted waveform can be written as [11]

(1) $$\begin{array}{cc} s_{k}\left(nT\right) & =\sqrt{E_{s}}\omega_{k}\left(nT\right)\\ \; & =\sqrt{\frac{E_{s}}{2^{SF}}}e^{j2\pi \left[\left(k+n\right)\;\mathrm{mod}\;2^{SF}\right]\frac{n}{2^{SF}}}, \end{array}$$

where $T=\frac{1}{B}$ is the sampling period, $n=0,1,2,\ldots ,(2^{SF}-1)$ is the sample index at time nT, $E_{s}$ is the signal energy, and $\omega_{k}\left(nT\right)$ are the $2^{SF}$ orthonormal basis functions. The receiver demodulates the signal using the outputs of the correlator as [11]

(2) $$\sum_{n=0}^{2^{SF}-1}r_{k}\left(nT\right)\cdot \omega_{i}^{*}\left(nT\right)=\left\{\begin{array}{cc} \sqrt{E_{s}}+\phi_{i}, & i=k\\ \phi_{i}, & i\neq k \end{array}\right.,$$

where $r_{k}\left(\cdot \right)$ is the received signal, $\phi_{i}$ depicts a complex Gaussian noise process, and $\omega_{i}^{*}\left(nT\right)$ is the complex conjugate of the *i*-th basis function.

As shown by (2), the chirp signal demodulation is based on orthogonality properties. Thus, the detection of the symbols happens with the index selection of the basis waveform that has the highest correlation magnitude with respect to the received signal. Therefore, for a received waveform of $r_{k}\left(nT\right)$, the detected index symbol is computed as [11]

(3) $$\tilde{k}=\left\{i|{arg}_{i}max\left(|\delta_{k,i}\sqrt{E_{s}}+\phi_{i}|\right)\right\},$$

where |·| is the absolute value operator, $\delta_{i,k}=1$ for i=k and $\delta_{i,k}=0$ otherwise. We also must define γ as the signal-to-noise ratio (SNR) which is expressed as [11]

(4) $$\gamma =\frac{E_{s}}{T_{s}}\frac{1}{N_{0}B}=\frac{E_{s}}{N_{0}\cdot 2^{SF}}.$$

## 3. Bit Error Probability

### 3.1. Error Probability for AWGN channels

From (2), we can derive a random variable such that $\rho_{i}={\left|\phi_{i}\right|}_{i\neq k}$. Since $\phi_{i}$ is a complex zero-mean Gaussian noise process, then $\rho_{i}$ will be a Rayleigh random distributed variable such that [12]

(5) $$F_{\rho_{i}}\left(x\right)=1-exp\left[-\frac{x^{2}}{2\sigma^{2}}\right],$$

where $\sigma^{2}=\frac{N_{0}}{2}$ and $N_{0}$ is the noise spectral density. The symbol error probability is given such that

(6) $$P_{e|k}=Pr\left[\max_{i,i\neq k}\left(\rho_{i}\right)>\beta_{k}\right],$$

where $\beta_{k}=\left|\sqrt{E_{s}}+\phi_{k}\right|$ follows a Rician distribution with shape parameter $K=\frac{E_{s}}{2\sigma^{2}}=\frac{E_{s}}{N_{0}}$. In this way, the probability density function of $\beta_{k}$ can be written as

(7) $$f_{\beta_{k}}\left(y\right)=\frac{y}{\sigma^{2}}\exp\left[-\frac{\left(y^{2}+E_{s}\right)}{2\sigma^{2}}\right]I_{0}\left(\frac{y\sqrt{E_{s}}}{\sigma^{2}}\right).$$

The random variable $\rho =\max\left(\rho_{i}\right){|}_{i\neq k}$ is defined as the maximum of ($2^{SF}-1$) i.i.d. Rayleigh random variables. Since the ($2^{SF}-1$) variables are independent, the cumulative distribution function for ρ can be given as [12]

(8) $$F_{\rho }\left(x\right)={\left[1-exp\left[-\frac{x^{2}}{2\sigma^{2}}\right]\right]}^{2^{SF}-1}.$$

Using (6)–(8), and equally probable symbols, we can express the average bit error probability $P_{b}$ as

(9) $$P_{e|k}=\int_{0}^{\infty }\left[1-{\left[1-\exp\left[-\frac{y^{2}}{2\sigma^{2}}\right]\right]}^{2^{SF}-1}\right]f_{\beta_{k}}\left(y\right)dy.$$

Expression (9) is the average bit error rate probability for AWGN channels. We can further simplify (9) using the Newton’s binomial identity

(10) 1−(1−ex)N=∑q=1NNq(−1)q+1exq,

then (9) can be written as

(11) Pe|k=∑q=12SF−1(−1)q+12SF−1q∫0∞e−qy22σ2fβk(y)dy.

Now, if we replace (7) into (11), we have

(12) Pe|k=∑q=12SF−1(−1)q+12SF−1qe−Es2σ2qq+1×∫0∞yσ2I0Esyσ2e−(q+1)y2+Esq+12σ2dy.

Fortunately, the integral in (12) can be computed as

(13) $$\int_{0}^{\infty }\frac{y}{\sigma^{2}}I_{0}\left(\frac{\sqrt{E_{s}}y}{\sigma^{2}}\right)e^{-\frac{\left(q+1\right)y^{2}+\frac{E_{s}}{q+1}}{2\sigma^{2}}}dy=\frac{1}{1+q},$$

Then, using (4), we find a closed-form expression for the error probability given the symbol *k* [12]

(14) Pe|k=∑q=12SF−1(−1)q+1q+12SF−1qexp−qq+1γ·2SF.

Then, according to [12], we finally find the average bit error rate such that

(15) Pb=2SF−12SF−1∑q=12SF−1(−1)q+1q+12SF−1qexp−qq+1γ·2SF.

### 3.2. Error Probability for Nakagami-*m* Channels

We first consider that a random variable α∼Gamma(k,θ). Then, we relate the Nakagami-m parameter to this distribution such that k=m and θ=Ω/m. In the case of Nakagami-*m* channels, the correlation output at the demodulator is given as

(16) $$\sum_{n=0}^{2^{SF}-1}r_{k}\left(nT\right)\cdot \omega_{i}^{*}\left(nT\right)=\left\{\begin{array}{cc} \sqrt{\alpha E_{s}}+\phi_{i}, & i=k,\\ \phi_{i}, & i\neq k \end{array}\right.$$

where $\sqrt{\alpha }$ follows a Nakagami-m distribution. Accordingly, (6) can be modified to include the Nakagami-*m* random variable as

(17) $$P_{e|\alpha }=Pr\left[\rho >|\sqrt{\alpha E_{s}}+\phi_{i}|\right].$$

Following similar rationale as in (9), the bit error probability for Nakagami-*m* can now be expressed as

(18) $$P_{e|\alpha }=\int_{0}^{\infty }\int_{0}^{\infty }\left[1-{\left[1-e^{-\frac{y^{2}}{2\sigma^{2}}}\right]}^{2^{SF}-1}\right]\Psi_{m}\left(y,\alpha \right)dyd\alpha ,$$

where

(19) $$\Psi_{m}\left(y,\alpha \right)=f_{\beta_{k}|\alpha }\left(y\right)\frac{m^{m}}{\Gamma (m)}\alpha^{m-1}e^{-m\alpha },$$

and

(20) $$f_{\beta_{k}|\alpha }\left(y\right)=\frac{y}{\sigma^{2}}\exp\left[-\frac{\left(y^{2}+\alpha E_{s}\right)}{2\sigma^{2}}\right]I_{0}\left(\frac{y\sqrt{\alpha E_{s}}}{\sigma^{2}}\right).$$

Note that Ω=1 in the term $\frac{m^{m}}{\Gamma (m)}x^{m-1}e^{-mx}$ is the normalized channel power for Nakagami-*m* distribution. The expression in (18) is the average bit error rate probability for Nakagami-*m* channels in the integral form. A closed-form expression of (18) can be obtained following the same rationale in the steps of (10)–(13), so that

(21) Pe|α=∑q=12SF−1∫0∞∫0∞2SF−1q(−1)q+1e−qy22σ2×Ψm(y,α)dydα,

In the sequel, we must apply a change of variable of the form $z=y^{2}$ and perform the integration in order to get

(22) Pe|α=∑q=12SF−1∫0∞2SF−1q(−1)q+1e−γq2SFq+1(q+1)mmαm−1e−mαΓ(m)dα

finally, integrating with respect to the *x* variable, we can get the following expression

(23) Pe|α=∑q=12SF−1(−1)q+1q+12SF−1q1+qm(q+1)γ·2SF−m

where γ was defined in (4). Note that

(24) $$\lim_{m\to \infty }{\left[1+\frac{q}{m(q+1)}\gamma \cdot 2^{SF}\right]}^{-m}=\exp\left(-\frac{q}{q+1}\gamma 2^{SF}\right)$$

and for this case (23) coincides exactly with (14), that is, the Gaussian case is the limit when the Nakagami-*m* parameter tends to infinity.

Again, according to [12], we finally find the average bit error rate for Nakagami-m such that

(25) Pb=2SF−12SF−1∑q=12SF−1(−1)q+1q+12SF−1q1+qm(q+1)γ·2SF−m

### 3.3. Error Probability for Rayleigh channels

Our new expression (23) is general and valid for any positive *m* parameter. The symbol error probability for Rayleigh channels can be obtained for the particular case of m=1 in (23), thus

(26) Pe|α=∑q=12SF−1(−1)q+12SF−1q1+qq+1γ·2SF−1q+1

The Gaussian hypergeometrical function is defined as [19]

(27) 2F1−mbc;z=∑n=0m(−1)nmn(b)n(c)nzn,

where ${\left(\zeta \right)}_{n}$ is the Pochhammer symbol defined as

(28) $${\left(\zeta \right)}_{n}=\left\{\begin{array}{cc} 1 & ,n=0\\ \zeta (\zeta +1)\ldots (\zeta +n-1) & ,n>0 \end{array}\right.$$

We can rewrite (26) as

(29) Pe|α=1+−1−∑q=12SF−1(−1)q+12SF−1q1+qq+1γ·2SF−1q+1=1−∑q=02SF−1(−1)q2SF−1q11+q+q2SFγ.

Notice that

(30) $$\frac{{\left(\frac{1}{1+2^{SF}\gamma }\right)}_{q}}{{\left(\frac{1}{1+2^{SF}\gamma }+1\right)}_{q}}=\frac{\left(\frac{1}{1+2^{SF}\gamma }\right)\left(\frac{1}{1+2^{SF}\gamma }+1\right)\left(\frac{1}{1+2^{SF}\gamma }+2\right)\cdots \left(\frac{1}{1+2^{SF}\gamma }+q-1\right)}{\left(\frac{1}{1+2^{SF}\gamma }+1\right)\left(\frac{1}{1+2^{SF}\gamma }+2\right)\cdots \left(\frac{1}{1+2^{SF}\gamma }+q-1\right)\left(\frac{1}{1+2^{SF}\gamma }+q\right)}.$$

Except for the first term in the numerator, and the last term in the denominator, all the terms in the numerator and denominator are the same and therefore can be canceled. We can finally, write (30) as

(31) $$\frac{{\left(\frac{1}{1+2^{SF}\gamma }\right)}_{q}}{{\left(\frac{1}{1+2^{SF}\gamma }+1\right)}_{q}}=\frac{\left(\frac{1}{1+2^{SF}\gamma }\right)}{\left(\frac{1}{1+2^{SF}\gamma }+q\right)}=\frac{1}{1+q+q2^{SF}\gamma }.$$

If we consider that z=1 from (27) and use (31), we can write

(32) Pe|α=1−∑q=02SF−1(−1)q2SF−1q11+q+q2SFγ=1−2F11−2SF11+2SFγ2+2SFγ1+2SFγ;1=1−Γ(2SF)Γ2+γ·2SF1+γ·2SFΓ1+2SF+γ·22SF1+γ·2SF.

The average error bit probability for Rayleigh is derived such that [12]

(33) $$P_{b}=\frac{2^{SF-1}}{2^{SF}-1}\left[1-\frac{\Gamma \left(2^{SF}\right)\Gamma \left(\frac{2+\gamma \cdot 2^{SF}}{1+\gamma \cdot 2^{SF}}\right)}{\Gamma \left(\frac{1+2^{SF}+\gamma \cdot 2^{2SF}}{1+\left(\gamma \cdot 2^{SF}\right)}\right)}\right].$$

It is important to emphasize that our closed-form solution given in (33) is exact, whereas (33) in [11], although very accurate, provides an approximation.

The new expression in (33), obtained here, allows getting some intuition with respect the equivalent signal to noise ratio γ. Note that when γ→0, that is for very low values of the equivalent signal to ratio, the ratio given by $\frac{\Gamma \left(2^{SF}\right)\Gamma \left(\frac{2+\gamma \cdot 2^{SF}}{1+\gamma \cdot 2^{SF}}\right)}{\Gamma \left(\frac{1+2^{SF}+\gamma \cdot 2^{2SF}}{1+\left(\gamma \cdot 2^{SF}\right)}\right)}$ tends to 0 and therefore $P_{b}\to \frac{1}{2}$ (for SF>6), as expected. On the other hand, for high values of γ, the same ratio tends to 1 and therefore $P_{b}\to 0$.

### 3.4. Rician Fading

In the case of Rician fading channels, the correlation output at the demodulator is given as

(34) $$\sum_{n=0}^{2^{SF}-1}r_{k}\left(nT\right)\cdot \omega_{i}^{*}\left(nT\right)=\left\{\begin{array}{cc} \sqrt{\left(\alpha +\nu \right)E_{s}}+\phi_{i}, & i=k,\\ \phi_{i}, & i\neq k \end{array}\right.$$

where ν is related to the direct path component. Following similar rationale as in (9), the bit error probability for Rician can now be expressed as

(35) $$P_{e|\nu }=\int_{0}^{\infty }\int_{0}^{\infty }\left[1-{\left[1-e^{-\frac{y^{2}}{2\sigma^{2}}}\right]}^{2^{SF}-1}\right]\Psi_{r}\left(y,\alpha \right)dyd\alpha ,$$

where

(36) $$\Psi_{r}\left(y,\alpha \right)=f_{\beta_{k}|\alpha }\left(y\right)\frac{\alpha }{\sigma_{r}^{2}}e^{-\frac{-\alpha^{2}+\nu^{2}}{2\sigma_{r}^{2}}}I_{0}\left(\frac{\nu \alpha }{\sigma_{r}^{2}}\right).$$

where $f_{\beta_{k}|\alpha }\left(\cdot \right)$ is given in (20), $\nu^{2}$ is the direct component power, $\sigma_{r}^{2}$ is the variance of the Rice distribution. We introduce the variable κ as the shape parameter which is the ratio of the power contributions by line-of-sight path to the remaining multi-paths, and Ω as the scale parameter related with the total power received in all paths. Furthermore, we can establish the following relations

(37) $$\begin{array}{cc} \nu^{2} & =\frac{\kappa }{\kappa +1}\Omega ,\\ \sigma_{r}^{2} & =\frac{\Omega }{2(1+\kappa )}. \end{array}$$

Expression (35) is the average bit error rate probability for Rician channels in the integral form. A closed-form expression of (35) can be obtained following the same rationale in the steps of (10)–(13), and substituting (37) so that

(38) Pe|ν=∑q=12SF−1(−1)q+11+q+2SFqγΩ1+κ2SF−1q×exp−γ2SFqκΩ1+κ+q1+κ+γ2SFΩ

Note, when Ω=1, we have that

(39) $$\begin{array}{cc} \; & \lim_{\kappa \to \infty }\left[\exp\left(-\frac{\gamma 2^{SF}q\kappa \Omega }{1+\kappa +q\left(1+\kappa +\gamma 2^{SF}\Omega \right)}\right)\right]=\exp\left(-\frac{q}{q+1}\gamma \cdot 2^{SF}\right), \end{array}$$

and

(40) $$\begin{array}{cc} \; & \lim_{\kappa \to \infty }\left[\frac{{\left(-1\right)}^{q+1}}{1+q+\frac{2^{SF}q\gamma \Omega }{1+\kappa }}\right]=\frac{{\left(-1\right)}^{q+1}}{q+1} \end{array}$$

which agrees with the limit case given in (14) for AWGN scenario when the Rician shape parameter tends to infinity. On the other hand, we have that

(41) $$\begin{array}{cc} \; & \lim_{\kappa \to 0}\left[\exp\left(-\frac{\gamma 2^{SF}q\kappa \Omega }{1+\kappa +q\left(1+\kappa +\gamma 2^{SF}\Omega \right)}\right)\right]=1, \end{array}$$

and

(42) $$\begin{array}{cc} \; & \lim_{\kappa \to 0}\left[\frac{{\left(-1\right)}^{q+1}}{1+q+\frac{2^{SF}q\gamma \Omega }{1+\kappa }}\right]=\frac{{\left(-1\right)}^{q+1}}{1+q+2^{SF}q\gamma } \end{array}$$

which agree with the limit case in the (26) of Rayleigh scenario when the Rician shape parameter tends to zero.

The average error bit probability for Rice case is derived such that [12]

(43) Pb=2SF−12SF−1∑q=12SF−1(−1)q+11+q+2SFqγΩ1+κ2SF−1q×exp−γ2SFqκΩ1+κ+q1+κ+γ2SFΩ.

## 4. Numerical Results

**Figure 2:**
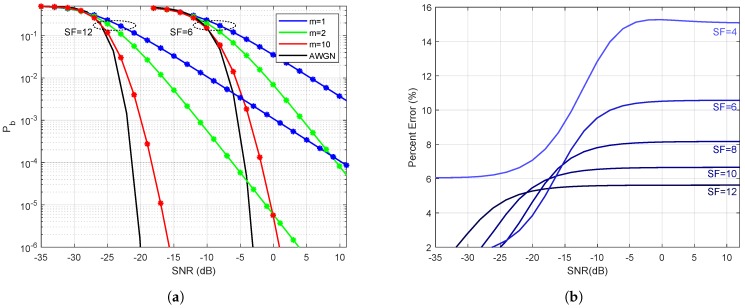
(**a**) Bit Error Rate for Nakagami-*m* and Rayleigh cases. The continuous blue curve is given by (26) (Rayleigh Integral) and the blue stars are computed using (33) (Rayleigh Exact). The green and red curves are the BER for the Nakagami-*m* case, the continuous and star lines are given by (18) (Nakagami-*m* Integral) and (23) (Nakagami-*m* Exact), respectively. Analytic AWGN curve is provided for reference. (**b**) Percentage error between the Rayleigh BER approximation in [11] and our *exact* analytical solution given in (33).

In this section, we will evaluate the numerical expressions and compare the results. Figure 2a presents comparisons of the derived Nakagami BER given in (23) and (33) versus the theoretical BER performance solved numerically using the integrals in (18) and (26), respectively. We choose SF∈{6,12} as the lowest and highest spread factor values to produce two sets of curves and each group with m∈{1,2,10}. As shown in Figure 2, the expressions in (23) and (33) are indistinguishable from (18) and (26). Also, note that as *m* increases, the bit error rate curve gets very close to the AWGN curve, as expected. With SF =12 and m=10, there is a gain of almost 30 dB for a BER of $10^{-4}$ in a multipath environment.

Figure 2b shows the percentage error of the difference between the Rayleigh BER approximation in [11] and (33). The difference can vary from 6% (when SF=12) to 15% (when SF=4).

Next, we focus on the accuracy of the results given by numerical methods considering integrals and a finite series. We calculated the percentage error between on (18) and (23). The data is summarized in the Table 1. The data was generated such that parameters assume SNR(dB)∈{−30,−10,10}, SF∈{12,10,8,6,4}, and m∈{1,3,5}. The numbers in the table are tiny, and therefore, it is necessary to multiply by a factor of $10^{-6}$ to get the percentage error.

**Figure 3:**
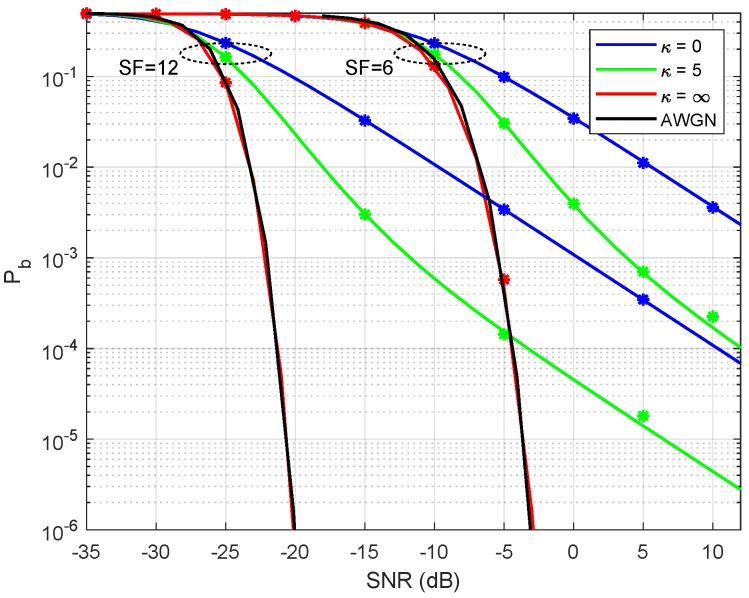
Bit error rate for the Rician case. The continuous lines is given by (38) and stars are given by the Matlab simulations. Analytic AWGN curve is provided for reference.

Figure 3 presents the derived BER in (38) versus simulation for Rician environments. Again, we choose SF∈{6,12} as the lowest and highest spread factor values to produce two sets of curves and each group with κ∈{0,5,∞}. As shown in the figure, the expression in (38) superposes with the discrete values found through simulation. Note that simulated and theoretical curves are almost indistinguishable. Also, note that as κ tends to infinity, the bit error rate curve gets very close to the AWGN curve, as expected.

## 5. Application Case

Since the signal to noise ratio γ in (23) is related to the maximum link distance, we can compute this distance in a system using the LoRa technology where the communication link is under the Nakagami-*m* distribution.

We choose the device SX1272 [18] as reference. For instance, the datasheet report that the receiver sensitivity is $P_{RX}=-137$ dBm for B=125 kHz and SF=12. Using (23), we can set a target bit error probability of $P_{b}=10^{-4}$ and analyze what is the impact of the Nakagami-*m* factor in the maximum link distance.

We choose Okumura-Hata [20] as our path-loss model with urban area parameters. The heights of the base station and mobile stations are 40 meters and 1 m, respectively. The system operates in the 900 MHz frequency, which agree with the limits presented in Table 2.

**Figure 4:**
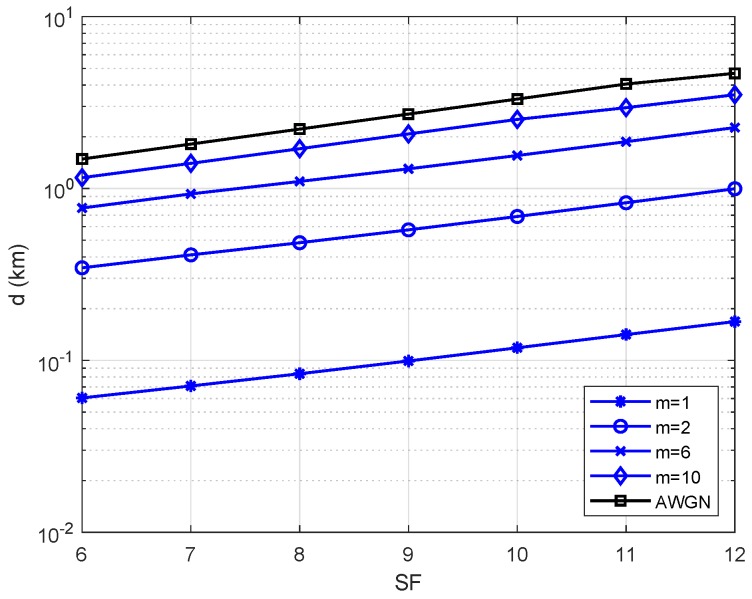
Maximum distance using the Okumura-Hata path-loss model for an urban area and a bit error probability of $P_{b}=10^{-4}$.

Figure 4 presents the maximum link distance for different values of SF and *m*. When we compare these curve against the AWGN curve (no fading case) given in (14), we notice that the distance decreases from 5 km to 200 m for SF=12, which indicates a loss of about 96% of link range. For scenarios where the Nakagami-*m* parameter ranges from 2 to 6, the link range loss reduces to 80% and 60%, respectively. For m=10, the performance becomes closer to the AWGN performance.

## 6. Conclusions

In this paper, we derived new expressions to assess the BER performance of CSS in the essential fading scenarios, i.e., Rayleigh, Rician, and Nakagami-*m* fading channels. All the results are in terms of LoRa parameters such as spreading factor SF, bandwidth *B*, and symbol to noise ratio $E_{s}/N_{0}$. The derived bit error probability expressions will be handy for any IoT provider that aims to predict the network coverage for different scenarios where the fading may vary. Also, we have addressed the limiting cases where m→∞ (Nakagami parameter) and κ→∞ (Rice parameter), showing that they converge to the non-fading case or the case under the influence only of the additive white Gaussian noise. All the expressions have been validated by simulation and when applicable, compared with previous results.

## Figures and Tables

**Table 1:** Table with percentage error values between (18) and (23). The value of each cell corresponds to the number times $10^{-6}$ %.

SNR = −30 dB					SNR = −10 dB					SNR = 10 dB				
	m	1	3	5		m	1	3	5		m	1	3	5
SF					SF					SF				
**12**		0.7	2	0.0	**12**		0.5	5.0	4.0	**12**		2.1	11.0	4.0
**10**		1.5	8.0	0.1	**10**		2.2	1.0	1.2	**10**		0.2	7.8	80.0
**8**		65.6	1.9	1.5	**8**		0.1	0.2	2.6	**8**		23.4	0.6	13.3
**6**		25.4	0.7	0.2	**6**		2.7	2.3	1.9	**6**		0.9	4.6	3.3
**4**		21.7	0.4	0.7	**4**		0.8	13.0	4.2	**4**		2.4	1.2	3.5

**Table 2:** Regions and Operating Frequencies for LoRa devices [18].

Region	Frequency (MHz)
EU	863–870
US	902–928
AU	915–928
CN	779–787 and 470–510
